# Metabolomics Coupled with Multivariate Data and Pathway Analysis on Potential Biomarkers in Gastric Ulcer and Intervention Effects of *Corydalis yanhusuo* Alkaloid

**DOI:** 10.1371/journal.pone.0082499

**Published:** 2014-01-15

**Authors:** Li Tianjiao, Wang Shuai, Meng Xiansheng, Bao Yongrui, Guan Shanshan, Liu Bo, Chen Lu, Wang Lei, Ran Xiaorong

**Affiliations:** 1 Liaoning University of Traditional Chinese Medicine, Dalian, P. R. China; 2 Agilent Technologies Co, Ltd., Beijing, P. R. China; 3 Liaoning University of Traditional Chinese Medicine-Agilent Technologies Modern TCM and Multi-omics Research Collaboration Lab, Dalian, P. R. China; The University of Kansas Medical Center, United States of America

## Abstract

Metabolomics, the systematic analysis of potential metabolites in a biological specimen, has been increasingly applied to discovering biomarkers, identifying perturbed pathways, measuring therapeutic targets, and discovering new drugs. By analyzing and verifying the significant difference in metabolic profiles and changes of metabolite biomarkers, metabolomics enables us to better understand substance metabolic pathways which can clarify the mechanism of Traditional Chinese Medicines (TCM). *Corydalis yanhusuo* alkaloid (CA) is a major component of Qizhiweitong (QZWT) prescription which has been used for treating gastric ulcer for centuries and its mechanism remains unclear completely. Metabolite profiling was performed by high-performance liquid chromatography combined with time-of-flight mass spectrometry (HPLC/ESI-TOF-MS) and in conjunction with multivariate data analysis and pathway analysis. The statistic software Mass Profiller Prossional (MPP) and statistic method including ANOVA and principal component analysis (PCA) were used for discovering novel potential biomarkers to clarify mechanism of CA in treating acid injected rats with gastric ulcer. The changes in metabolic profiling were restored to their base-line values after CA treatment according to the PCA score plots. Ten different potential biomarkers and seven key metabolic pathways contributing to the treatment of gastric ulcer were discovered and identified. Among the pathways, sphingophospholipid metabolism and fatty acid metabolism related network were acutely perturbed. Quantitative real time polymerase chain reaction (RT-PCR) analysis were performed to evaluate the expression of genes related to the two pathways for verifying the above results. The results show that changed biomarkers and pathways may provide evidence to insight into drug action mechanisms and enable us to increase research productivity toward metabolomics drug discovery.

## Introduction

Gastric ulcer is a widespread disease that afflicts many people all over the world due to its higher and higher morbidity. According to the statistics of 2005, the incidence of gastric ulcer was up to 80%, especially western world. It has 40–80% of recurrent frequency all over the world. Gastric ulcer in humans occur frequently because of various endogenous and exogenous factors such as stress, smoking, nutritional deficiencies, hydrochloric acid, pepsin, *Helicobacter pylori*, non-steroidal anti-inflammatory drug use (NSAIDs), alcohol and infection [1]. While these factors have been thought to be involved in the pathogenesis of gastric ulcers, the mechanism of ulcer formation is not yet precisely understood [2], [3]. TCM has gained increasing acceptance worldwide in recent years and is generally considered as being natural and harmless [4], [5]. Therapies collectively called TCM are commonly used to treat gastric ulcer, which includes Chinese herbal medicine and prescription etc.

QZWT prescription composed of *Corydalis yanhusuo*, *Radix Glycyrrhizae* and *Radix Bupleuri* ect. have been extensively used to cure gastric ulcer for centuries in China, owing to its significant therapeutic performance in clinical application [8]–[10]. We purified CA from plants “Corydalis yanhusuo W.T. Wang” with the purity of 92%. The chemical constituents of *Corydalis yanhusuo* have been investigated in our previous study. Tetrahydropalmatine, corydaline, protopine et al were the biological activities of *Corydalis yanhusuo*. The structures of the constituents were examined. CA is acknowledged to be the major active component in *Corydalis yanhusuo* [6], and demonstrated to have antiulcer effect being used in Chinese clinical practice for many years [7]. It has also been reported that CA possesses anti-inflammatory effect [8], [9]. However, the detailed molecular mechanism of CA in treating gastric ulcer is not well understood.

To explain the action mechanism of drugs, metabolomics methodology has been widely used [10]. Metabolomics is an important component of systems biology, especially in determining the global metabolic profile by detecting thousands of small and large molecules in various media ranging from cell cultures to human biological fluids such as urine, saliva, and blood [11], [12], [13]. It has a great impact in investigation of discovering biomarkers, and identifying perturbed pathways due to disease or drug treatment [14]. By analyzing and verifying the specific early biomarkers of a disease, metabolomics enables us to better understand substance metabolic pathways which can clarify the mechanism of action [15].

Recent advances of instrumentation and computation have enabled the simultaneous analysis of a large number of metabolites. HPLC coupled with MS has been proven to be an effective combination for metabolites identifications and quantifications due to its excellent resolution and sensitivity. The aim of current study was to obtain a systematic view to dissect the mechanism of CA as an effective treatment for gastric ulcer. The specific and unique biochemical pathways of drug efficacy can be identified, when coupled with multivariate data analysis techniques. The purpose of this study is to identify multiple metabolites that could facilitate the understanding of the action mechanism of CA and aid their incorporation into future improvement of TCM therapy.

## Materials and Methods

### 2.1 Ethical Statement

All experiments were performed in accordance with the approved animal protocols and guidelines established by Medicine Ethics Review Committee for animal experiments of Liaoning University of Traditional Chinese Medicine.

### 2.2 Animal Handling and Sample Preparation

Seven-week-old male SD rats weighing 200–250 g, were provided by the experimental animal centre of Dalian Medical University. The care and handling of rats were in accordance with the standard of Specific Pathogen Free. Gastric ulcer was induced in the rats according to the method in a previous report with a slight modification [16], [17]. Three days after the production of gastric ulcer, the rats were randomised into five groups: control, model, CA high dose group (32.4 mg/ml), CA middle dose group (10.8 mg/ml) and CA low dose group (3.6 mg/ml). All the rats, in groups were orally administered of the active group solution 1.5 ml once daily (model and control groups with saline) for 7 days. The rats were prohibited any food for 12 h before the experiments, but were allowed access to water freely.

On the last day, rats were deeply anesthetized and then sacrificed. Blood was collected, plasma and serum were separated via centrifugation at 3000 rpm for 15 min at 4°C. The plasma samples were collected and stored at −80°C flash frozen in liquid nitrogen until metabolomics analysis were performed. Then, the stomachs were cut along the greater curvature, washed with saline. The area of ulcer was measured by a compass to measure ulcer index. The area of ulcer equals to the width of the ulcer times the length of the ulcer. For histological evaluation, gastric tissue samples were fixed in neutral buffered formalin for 24 h. Stomach sections were dehydrated with graded ethanol, passed through xylene, and embedded in paraffin. Paraffin sections (5 mm thickness) were stained with hematoxylin/eosin (HE). The other gastric ulcerated tissues were rapidly removed and frozen in liquid nitrogen until the extraction of total tissue RNA.

### 2.3 Metabolic Profiling

#### 2.3.1 Chromatography

Chromatography was performed using an Agilent 1100 series HPLC system equipped with quaternary pump, online degasser, autosampler, and thermostated column compartment. The injection volume was fixed at 4 µL. All the samples were maintained at 4°C during the analysis. The separation was performed on a 4.6*100 mm, ZORBAX SB-C18 column (Agilent, USA). The column temperature was set at 45°C. The mobile phases were composed of 0.1% formic acid in water (solvent B) and 0.1% formic acid in acetonitrile (solvent A), the flow rate was set as 1 ml/min with split ratio 1∶3, the gradient was used as follows: a linear gradient of 70– 33% B over initial– 5.0 min, 33 –98% B over 5.0–12.0 min. The eluent was introduced to the mass spectrometer directly. After every 10 samples injecting, a pooled sample as the QC sample followed by a blank was injected in order to ensure the stability and repeatability of the LC-MS systems.

#### 2.3.2 Mass Spectrometry

For mass spectrometry, the Agilent 6220 TOF-MS with an electrospray ionization source (ESI) in negative mode was used. The flow rate of dying gas (N2) was set at 9 L/min. The nebulizer was set at 45 psi. The other optimal conditions were as follows: dying gas temperature of 350°C, fragment voltage of 120 V. Data were collected in the full-scan mode from m/z 50 to 1050 amu over 0–12 min. The MS data were collected in centroid mode.

#### 2.3.3 Multivariate data analysis

**Figure 1:**
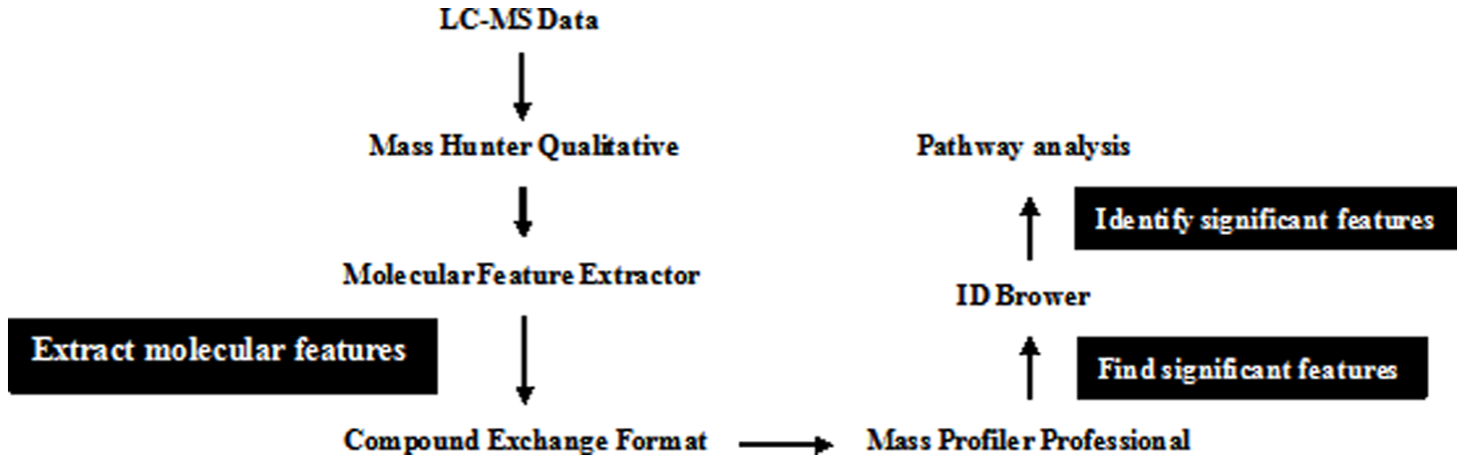
Data analysis procedure by MPP.

Data analysis procedure is shown in Fig. 1. The Molecular Feature Extractor (MFE) algorithm in the Mass Hunter Qualitative analysis software was used to extract molecular features—unidentified, untargeted compounds — in each of the data. The MFE algorithm looks for mass signals (ions) that are covariant in time, considers possible chemical relationships (isotopes, adducts, dimers, multiple charge states), and generates an extracted compound chromatogram and compound mass spectrum for each molecular feature. The extracted compound list for each file was exported as Compound Exchange Format (. cef) file for further Mass Profiler Professional (version B.2.00, Agilent) statistical analysis. The resulting feature files for each sample were processed by ANOVA and PCA analysis utilizing the MPP software, which aligned, normalized, visualized and filtered the molecular features (MFs), for further processing [18], [19], [20], [21]. Subsequently, hierarchical clustering (condition tree) was applied to the data files. Hierarchical cluster analysis is a statistical method to group samples unsupervised in different clusters or branches of the hierarchical tree. In this way, the relationships between the different groups are shown. The condition tree was displayed as a heat map. The identity of biomarkers with significant changes in the groups was determined by ID Browser features of MPP.

#### 2.3.4 Biomarkers Identification

The identification of potential biomarkers was determined by Q-TOF (Xevo G2). The MS collision energy is 35ev, and the data was obtained in the negative ion mode, x (V4. MassLn1) software was used for data analysis. The identities of the specific metabolites were confirmed by elements information comparison of their mass spectra using the elemental composition information provided by the software.

#### 2.3.5 Network and Pathway Analysis

MPP software was employed to all significant (folder change >2) up regulated and down regulated metabolites and related biological pathways. The potential markers identified were compared with the accurate mass charge ratio in some databases, including HMDB, KEGG, METLIN, LIPID MAPS and PUBCHEM, to discover related pathways. T-test and fold-alter were used to determine the statistical significance in the pathways. P value<0.05 and folder change>2 was considered to be criteria for statistically significant and would be selected.

### 2.6 Molecular Data

Total RNA was extracted from stomach tissues including control, model and CA groups using a TRIZOL reagent (Invitrogen, Carlsbad, CA, USA) per the manufacturer's instructions. cDNA was synthesized from total RNA (1 µg) using TransScript First-Strand cDNA Synthesis SuperMix kit (Beijing TransGen Biotech, China). Quantitative real time PCR (CFX96, BIO-RAD, USA) was performed using a TransStart™ Top Green qPCR SuperMix kit (Beijing TransGen Biotech, China). Primers used to amplify S1Pr1, S1Pr3, SphK1, Got2 and Fabp1 were from Invitrogen (S1Pr1: GenBank acc. no. NM_017301, S1Pr3: GenBank acc. no. XM_225216, SphK1: GenBank acc. no. NM_133386, Got2: GenBank acc. No. NM_013177.2, Fabp1: GenBank acc. no. NM_012556.2) and expression of these transcripts was quantified against the housekeeping gene β-actin, which was amplified using the primers 5′-TGGCACCACACTTTCTACAATGA-3′ and 5′-AGGGACAACACAGCCTGGAT-3′. Expression levels of target genes were analyzed using the CFX Manager system (BIO-RAD, USA).

### 2.7 Statistical Analysis

Data are expressed as mean ± SEM. SPSS 19.0 for Windows was used for the statistical analysis. The data were analyzed using the ANOVA, with p<0.05 set as the level of statistical significance.

## Result

### 3.1 Effect of CA on the acetic acid injected-induced gastric ulcer model

**Figure 2:**
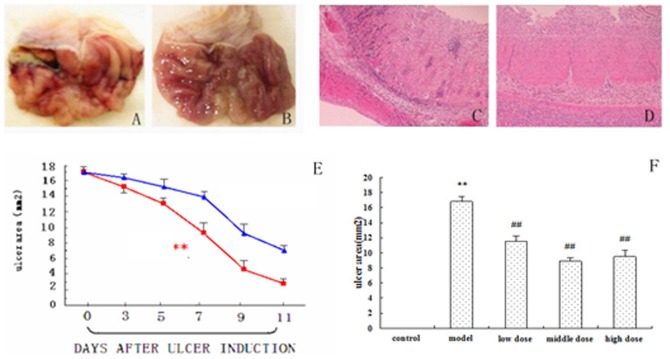
Macroscopic findings and histopathological observations. Note: A: model; B: control; C: model; D: control. E shows the time-course of changes in the area of the mucosal lesions between CA treated group and model group. Red line represent CA group; blue line represent model group. Effect of CA on healing of gastric ulcers on day 7 after ulcer induction are demonstrated in F.Notes: Data are the mean ± SD. ** Significantly different (p<0.01) from the control. ^##^ Significantly different (p<0.01) from the model.

The experimental model of acetic acid injected-induced gastric mucosal damage in rats is often employed to screen the compounds for anti-ulcer activity in that it serves as the leading cause of gastric ulcer in humans [22]. Acetic acid injected-induced intense gastric mucosal damage in the formation of ulcer in the model group (Fig. 2A) rats that has a significantly difference compared with the control group (Fig. 2B). Pathological observation was used to confirme the damage of acetic acid-induced in the superficial layers of gastric mucosa ulteriorly. Acetic acid-induced gastric ulcers (Fig. 2C) has an erosion effect to the mucosa, which was accompanied by muscle fracture and inflammatory cell infiltration in the layers compared with control (Fig. 2D). The results proved that the model of gastric ulcer was successfully reproduced. The results of the time-course showed in Fig. 2E illustrate that the area of ulcer in rats treated with CA remained significantly smaller when compared to the respective values in the model rats at seventh day, so we choosed the seventh day's samples for analysis. To evaluate the effects of CA, as demonstrated in Fig. 2F, the area of gastric ulcer in CA dose groups was significantly decreased compared with the model group (p<0.01). Our experimental results suggest that CA can effectively cure the gastric ulcer, particularly the middle dose group. It seems that there is a marked overlap among the neuronal pathogenetic pathways involved in ulcer genesis and depression. Therefore, it is not surprising that medication for the treatment of depressive episodes can also exert potent protective effect against gastric ulcer [23]. The reason why CA middle dose group have a better therapeutic effect than the high dose group may be CA high dose group has a role to inhibit nerve. The effect of CA was examined to further investigate the mechanism.

### 3.2 Metabolomic Study

#### 3.2.1 Acquisition and processing of metabolic profile data

**Figure 3:**
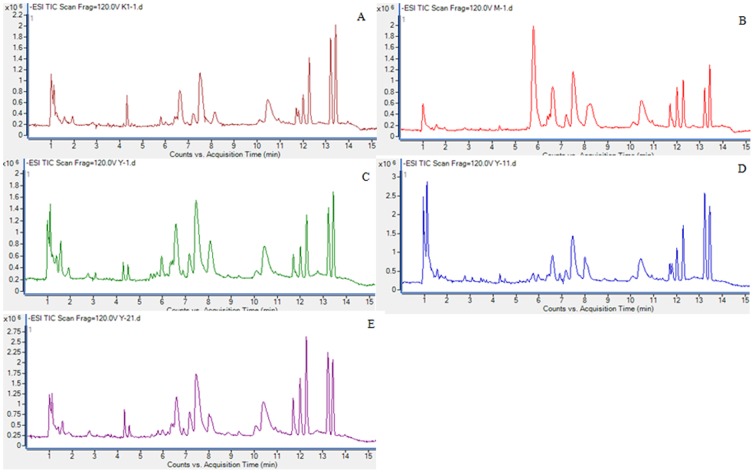
HPLC-MS TICs of plasma samples from each groups. Note: A: control; B: model; C: CA low dose group; D: CA middle dose group; E: CA high dose group.

**Figure 4:**
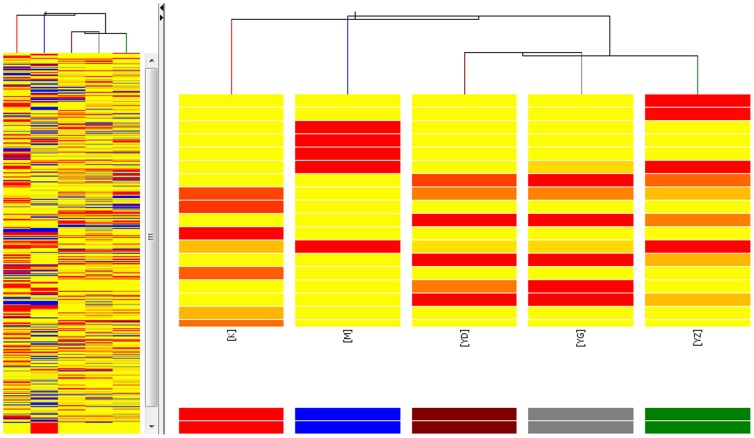
Unsupervised clustering analysis of the biomarkers in gastric ulcer. Note: The pseudo-colors indicate the relative metabolite level from increased (red) to decreased (blue) expressions with yellow representing unchanged.

**Figure 5:**
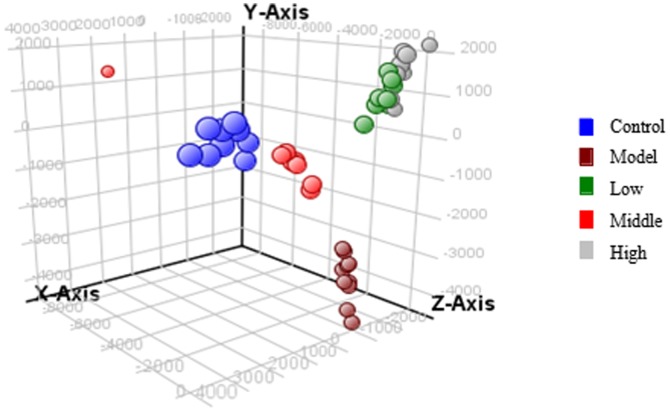
Principal component analysis (PCA) of each groups. Note: Each colored point represents a sample. The first, second and third principal components are displayed on the X, Y and Z-axis, respectively. These three components represent the largest fraction of the overall variability. Blue ball : control; brown ball: model; red ball: middle dose group; green ball: low dose group; gray ball: high dose group.

The representative total ion chromatograms(TIC) of plasma samples derived from control, model, and CA dose groups in negative modes are presented in Fig. 3 by using the optimal LC-MS conditions described above. Low molecular mass metabolites could be separated well in the short time of 15 min. In order to better visualize the subtle similarities and differences among these complex data sets, multiple pattern recognition methods were employed to phenotype the plasma metabolome of rats. Here, hierarchical clustering analysis and PCA were used to classify the metabolic phenotypes and identify the differenting metabolites. Hierarchical clustering analysis of metabolomics data showed distinct segregation between the control, model group and CA dose group (Fig. 4). In the PCA scores, each point represents an individual sample. The PCA results are displayed as score plots indicating the scatter of the samples, which indicate similar metabolomics compositions when clustered together and compositionally different metabolomes when dispersed. The PCA scores plot could divide the different plasma samples into different blocks, respectively, suggesting that the metabolic profiles have changed. With regard to information analyst of PCA in our experiment showed in Fig. 5, the control and model groups were significantly divided into two classes, indicating that the model of acetic acid-induced gastric ulcer was successfully reproduced. More subtle changes can be found by the pattern recognition approach-score plots of PCA. PCA results display that the model group was far away from the remaining four groups, indicating that changed metabolic pattern resulted from acetic acid-induced may be significantly different from others. The position of treatment group was near to the control group, suggesting that changed metabolic pattern was caused by CA. The results manifest that CA could change the abnormal metabolic status and may have a different treatment mechanism of acetic acid-induced gastric ulcer.

#### 3.2.2 Identification of potential biomarkers

**Figure 6:**
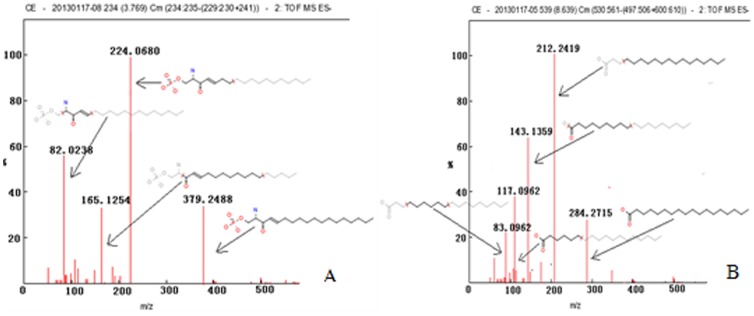
Q-TOF MS spectra of S1P (A) and stearic acid(B).

The small-molecule metabolites of significant differences (T-test, p<0.05) were searched by the software of MPP. The potential markers were identified by using the “ID browser” to search in Metlin database (http://metlin.scripps.edu/) and compared with the accurate mass charge ratio in some databases, including HMDB (http://www.hmdb.org/), KEGG (http://www.genome.jp/kegg/), LIPID MAPS (http://dev.lipidmaps.org:25424/), and PUBCHEM (http://pubchem.ncbi.nlm.nih.gov/). We can know the probable name of potential biomarkers through the first step. In the present study, 10 potential biomarkers were identified (Table 1). The precise molecular mass of compounds with significant changes in the groups was determined within measurement errors (<5 ppm) by Waters Xevo G2 QTOF, and meanwhile, the potential elemental composition, degree of unsaturation and fractional isotope abundance of compounds were obtained. The presumed molecular formula was searched in Chemspider (http://www.chemspider.com/), HMDB and other databases to identify the possible chemical constitutions, and MS/MS data were screened to determine the potential structures of the ions. Sphingosine-1-phosphate (S1P) and stearic acid were taken as examples to illustrate fragments of the structure and the appraisal process. The primary and secondary mass spectrometry information was analyzed by Masslynx (vision 4.1, waters) software, compared with database, and ion fragments of 379.2488 (C_18_H_38_NO_5_P) were shown in Fig. 6 A. The main fragment ions analyzed by MS/MS screening were m/z 224.080, 165.1254 and 82.0238, which could correspond to lost C_7_H_15_NO_5_P, C_11_H_17_O, C_4_H_4_NO respectively. Finally, it was speculated as S1P after refering and according to their polarity size. Meanwhile, ion fragments of stearic acid 284.2715 (C_18_H_36_O_2_) (Fig. 6 B) were 212.2419 (C_15_H_32_), 143.1359 (C_9_H_19_O), 117.0962 (C_6_H_13_O_2_) and 83.0962 (C_6_H_11_).

**Table 1:** The identified potential biomarkers and metabolic pathway between groups.

	RT	m/z	Molecular formula	metabolites	Metabolic pathway
1	1.018	336.3200	C_6_H_12_O_6_	D-glucose	glucuronidation
2	1.021	146.1051	C_6_H_14_N_2_O_2_	L-Lysine	Biotin metabolism
3	1.063	168.0284	C_5_H_4_N_4_O_3_	Uric acid	Folic acid network
4	1.128	88.0623	C_3_H_4_O_3_	Pyruvic acid	Glycolysis and gluconeogenesis
5	1.441	204.0904	C_11_H_11_N_2_O_2_	D-Tryptophan	Folic acid network
6	3.588	487.6012	C_28_H_41_NO_6_	Glycocholate	Fatty acid biosynthesis
7	4.964	346.2142	C_21_H_30_O_4_	corticosterone	Biosynthesis of aldosterone and cortisol
8	5.188	381.2643	C_18_H_40_NO_5_P	sphingosine-1-phosphate	Sphingolipid metabolism
9	6.132	286.4157	C_16_H_30_O_4_	hexadecanedioic acid	Fatty acid biosynthesis
10	9.363	284.2712	C_18_H_36_O_2_	stearic acid	Fatty acid biosynthesis

**Figure 7:**
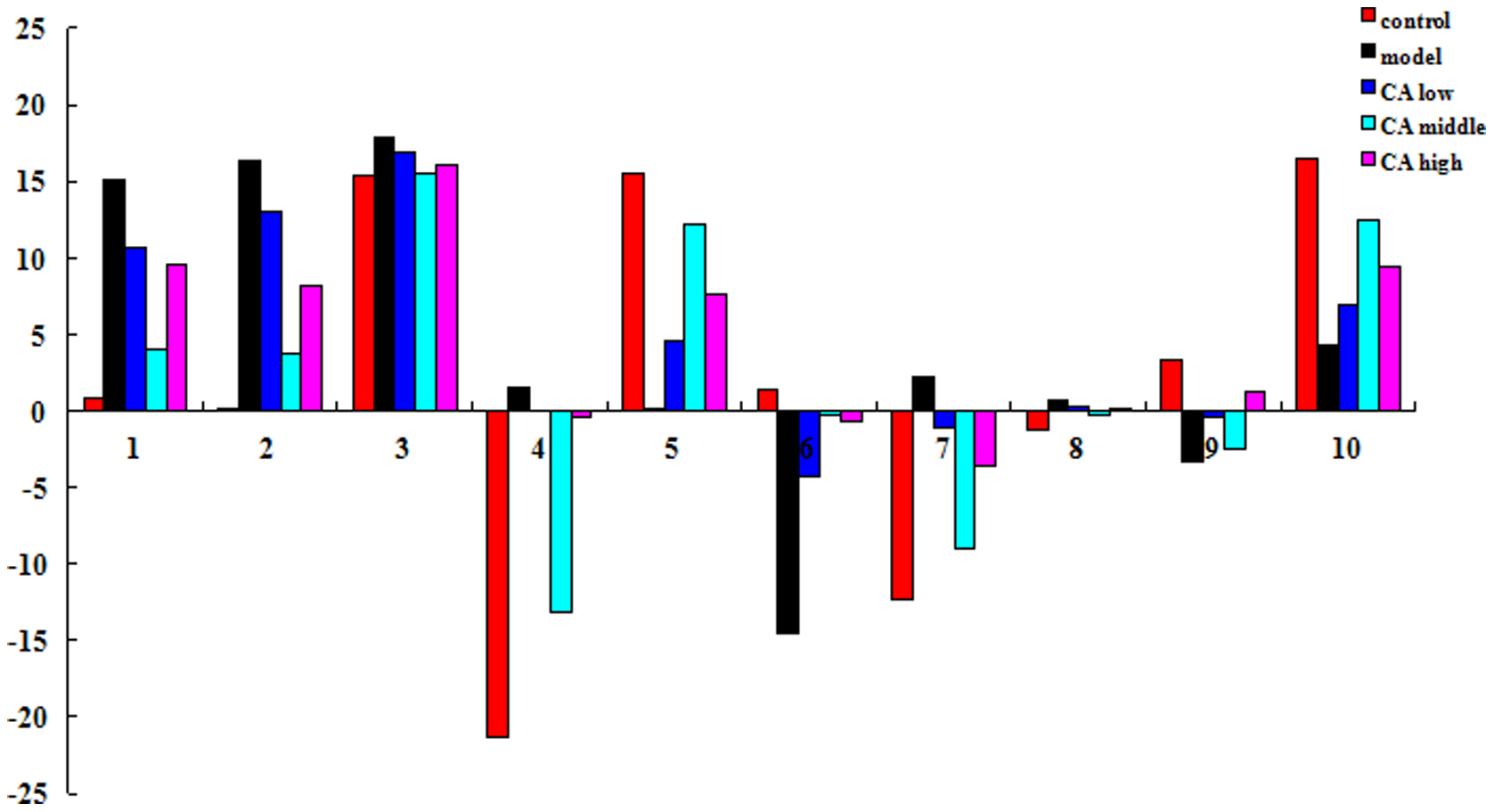
Differential expression levels (mean) of biomarkers in different groups.

**Figure 8:**
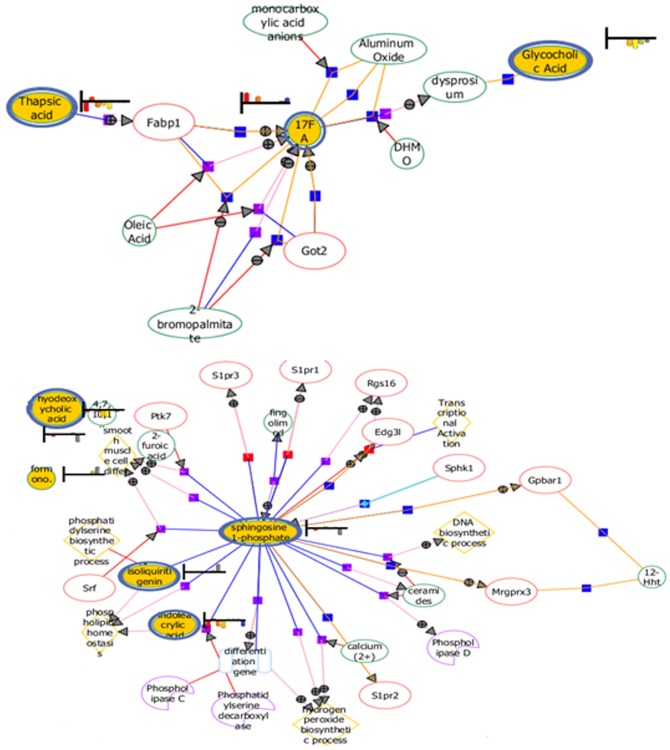
The network of gastric ulcer. Note: Yellow points in the fig represent biomarkers that have significant difference between the metabolites. The multi-point and multi-pathway involve in combined effect to the formation and healing of gastric ulcer.

The biomarkers described above were proved have close relationship with the formation and treatment of gastric ulcer. The significantly up regulated D-glucose, lysine, Uric acid, pyruvic acid, corticosterone, sphingosine-1-phosphate and the down regulated tryptophan, glycocholate, hexadecanedioic acid, stearic acid were observed in the model group compared with control group (Fig. 7). This difference of metabolites may denote their potential as targeted biomarkers for differentiating gastric ulcer and normal states. Monitoring changes of these metabolites may predict the development of gastric ulcer. The biomarkers 1, 2, 3, 4, 7, 8 were decreased after the treatment of CA, in contrary, the other biomarkers were increased. Additionally, in order to characterize antiulcer effects of CA more clearly, changes in the relative concentrations of target metabolites identified in different groups was analyzed, we have found that content of these key markers closer to normal group. The results indicate the mechanism for the treatment of gastric ulcers may be achieved through the regulation of these significantly markers and their interaction like Fig. 8. For example, stearic acid which called 17FA, has relationship with thapsic acid though the protein Fabp1 (fatty acid-binding protein 1). The network not only indicates the interaction between biomarkers, but also provides information of potential protein, genes, enzymes and biological processes. It contributes to the discovery of target during the occurrence and treatment of gastric ulcer and is conductive to the development of new drug to cure gastric ulcer.

### 3.3 Determination of mRNA levels to confirm the biomarkers

**Figure 9:**
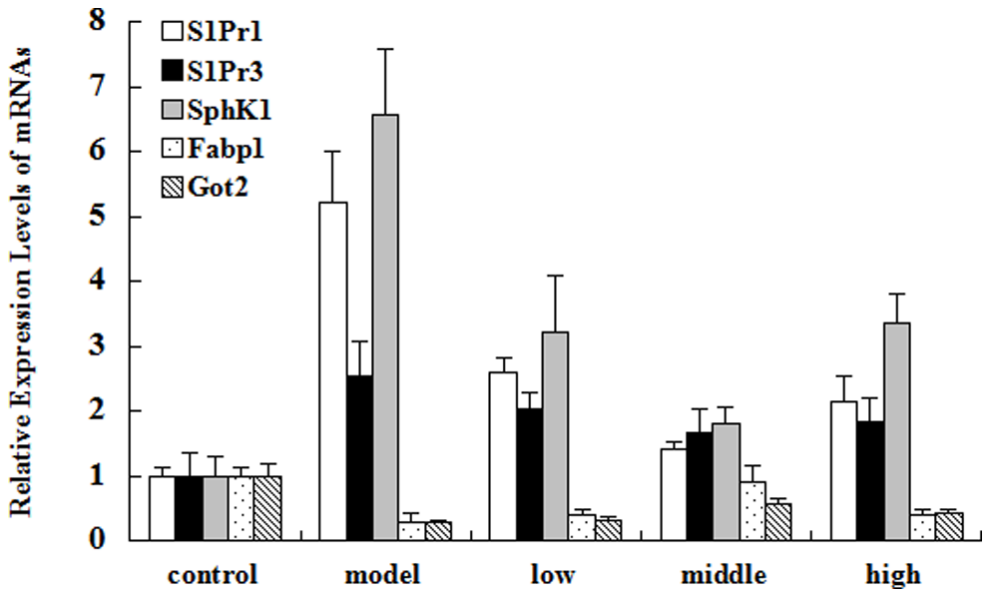
The expression level of mRNAs. Note: Abscissa represents the mRNAs of sphingolipid metabolism (including S1Pr1, S1Pr3 and SphK1) and fatty acid metabolism (including fabp1 and got2). The method of relative quantitative analysis was used to compare the gene expression in each group. The ordinate represents the relative expression levels of mRNAs in the basis of control group. Quantitative PCR results represent mean ± SEM of three independent experiments.

To confirm our metabolomics findings, we need some molecular data, so we identified 5 mRNAs which are related to the 4 potential biomarkers and 2 metabolic pathways with RT-PCR. Sphingolipid metabolism, including S1Pr1, S1Pr3 and SphK1 were examined as showed in Fig. 8. The results are summarized in Fig. 9. The mRNA level of S1Pr1, SIPr3 and SphK1 were significantly upregulated in the model group, the expression levels were 5.21, 2.54, 6.57 times compared to the control group, which was in agreement with our previous findings and data. After CA treatment, the expression levels of S1Pr1, S1Pr3 and SphK1 were back to basal level. S1P is formed by two kinases, sphingosine kinase 1 and 2 (SphK1 and SphK2), but no differences were observed in SphK2 expression among all the groups (data not shown), the result was consistent with our network findings. Here, we can explain a potential mechanism of CA in treating gastric ulcer by blocking S1P increasing. We also found an decreased expression of Fabp1 and Got2 in model group (Fig. 9), compared with control group. But CA does groups were near to the control group, which confirmed that the therapeutic effect of CA was related to fatty acid metabolism from molecular level.

### 3.4 Pathway Analysis

More detailed analysis of pathways and networks influenced by gastric ulcer was performed by MPP. The pathways obtained shows in Table 2. We use the high-quality KEGG metabolic pathways as the backend knowledge base to identify the most relevant pathways, such as sphingolipid metabolism, tricarboxylicacidcycle acid cycle, biotin metabolism and so on, in which 7 unique pathways (listed in Table 1) for the model group was identified. Potential biomarkers related to folic acid metabolism, fatty acid metabolism, and sphingolipid metabolism pathways were also conformed. Of the 6 distinct metabolites identified from these pathways, many are in various stages of progress of the gastric ulcer. Some significantly changed metabolites like glycocholate, hexadecanedioic acid, and stearic acid have been found and used to explain the fatty acid mechanism. These results suggest that these target pathways show the marked perturbations over the formation of gastric ulcer and could contribute to the development of gastric ulcer.

**Table 2:** The pathways obtained by MPP.

Pathway	C-M	C-M-C
SDIS Susceptibility Pathway	+	+
Folic Acid Pathway	+	+
Selenium Pathway	+	+
Biosynthesis of aldosterone and cortisol	+	+
Network Targets and Regulators	+	+
Expand Interactions	+	+
Direct Interaction	+	+
Glucuronidation		+
Polyol Pathway		+
D-Glocuse-INS-RXRA		+

Notes. C-M: control vs model; C-M-C:C-M vs *Corydalis yanhusuo* alkaloid dose groups.

## Discussion

Gastric ulcers in humans recur frequently, and the difficulty in treating them is indicated by the adage “Once an ulcer, always an ulcer” [24]. Many factors can increase the incidence of gastric ulcer, but the mechanism has not been understood clearly. Therefore, the effectiveness of drug treatment depends not only on the decrease of damaging factors, but also on the changed metabolites that regulate the metabolism pathway. In particular, the discovery of biomarkers that predict the risk of gastric ulcer will provide an opportunity to diagnose and permit pharmacological treatment timely. QZWT was used to cure gastric ulcer for many years in Asia although its mechanism remains unclear. Metabolomics coupled with multivariate data tools that simultaneously quantify thousands of metabolites in a living organism was used to analyze the biomarkers in gastric ulcer [25]. Additionally, understanding of biomarkers has sparked new interest in the fields of drug discovery programmes and disease monitoring, providing valuable in-sights about complex disease mechanisms [26]. This study was therefore designed to further elucidate the underlying mechanism of CA on gastric ulcer regulation from the metabolic pathways in a global view.

The model of gastric ulcer in rats was successfully reproduced. Plasma samples were analyzed by HPLC/ESI-TOF-MS and multivariate statistical analysis. The results showed that the area of the ulcer and dynamic metabolic profiles after CA treatment were closed to the control group, demonstrating that CA had therapeutic efficacy. According to metabolomics analysis, 10 potential biomarkers and 7 related metabolic pathways were identified in our study. The significantly down regulated D-glucose, lysine, uric acid, pyruvic acid, corticosterone, sphingosine-1-phosphate and the up regulated tryptophan, glycocholate, hexadecanedioic acid, stearic acid were observed in the CA group compared with model group. In addition, folic acid metabolism, fatty acid metabolism,and sphingolipid metabolism and many other metabolism were confirmed to have an impact on gastric ulcer. We have determine the expression of mRNAs related to sphingolipid metabolism and fatty acid metabolism to validate the mechanism. Many other potential proteins, genes, enzymes and bioprocess closed to other pathways need future experiments to validate.

By analyzing and verifying the specific early biomarkers of a disease, metabolomics enables us to better understand pathological processes and substance metabolic pathways. We believe that the biomarker and pathway analyses have great potential to explore and clarify the therapeutic action of TCM. In current study, we have characterized biomarker interaction networks involve proteins, genes, enzymes and bioprocess as shown in Fig. 8. S1P act extracellularly as a ligand for its specific receptors-S1PRs, is now recognized as regulator of many physiological and pathophysiological processes, including inflammatory disorders, such as rheumatoid arthritis, inflammatory bowel disease, and sepsis [27], [28]. Inflammation that occurs in the mucosal of gastrointestinal tract, thereby, causes gastrointestinal ulcer [29]. Our results show that CA can decrease the expression of S1P and its receptors, including S1Pr1 and S1Pr3, to relieve inflammation problems to relieve the formation of gastric ulcers [30]. S1P is formed by SphK1 and SphK2 [31]. SphK1 can active the NF-κB pathway which initiated by the major inflammatory signaling molecule TNF-α. In brief, NF-κB and TNF-α is closely related to form and heal gastric ulcer [32], [33]. We also found that the deficiency of SphK1(not SphK2) significantly inhibits gastric ulcer, indicating that SphK1 may play a pivotal role in gastric ulcer. Thus, the sphingolipid metabolism may be a viable target for treating gastric ulcer.

Stearic acid, glycocholate and hexadecanedioic acid changed cause fatty acid metabolism disorder closing to the incidence and rehabilitation of gastric ulcer [34], [35]. Fatty acids, including stearic acid etc, commonly viewed as the source of energy, have attracted interest for research and public health, due to their effects on human health and diseases. Fatty acids are beneficial to the health-promoting. Stearic acid, glycocholate and hexadecanedioic are regulated by Fabp1, the enzyme of fatty acid-binding protein 1. In analysis of RT-PCR, the low expression of Fabp1 in model group suggests that Fabp1 activity inhibiting may reduce stearic acid, glycocholate and hexadecanedioic acid, and lead to fatty acid metabolism disorder. Therefore increased the inflammatory response and mitochondrial dysfunction and promote ulcer formation. However, CA can balance this disorder through increasing the expression of Fabp1 [36]. Glutamic-oxaloacetic transaminase 2 (Got2) is an important enzyme in the tricarboxylicacidcycle acid cycle (TCA cycle). The severely inhibition of TCA caused by the decreased of Got2 will contribute to formation of gastric ulcer. The metabolites of amino acids such as tryptophan and its metabolites in vivo have a extensive role in tryptophan metabolism. The most important is that tryptophan metabolism disorders can cause TCA disorder. TCA play a role in healing gastric ulcer [37]. Down-regulation of Got2 mRNA expression in model group and up-regulation in CA groups were previously demonstrated in our result. All these data clearly indicate that the molecular mechanism of CA treating gastric ulcer was closely correlated with its balance effects on TCA. These results implicate the CA effects may be mediated through protein, enzymes, and metabolism pathway. It provided strong evidence that the hypnotic effect of CA occurred at the level of global metabolomics.

Metabolomics is one functional level tool being employed to investigate the complex interactions of metabolites with other metabolites (metabolism) but also the regulatory role metabolites provide through interaction with genes, transcripts and proteins. Potential roles for metabolomics in the clinical trials of gastric ulcer include biomarker discovery and validation, molecular target discovery, therapy decisions. Metabolomics has already shown promise in identifying metabolite based biomarkers in gastric ulcer as biochemical profiling tools to provide important insight into the biology of gastric ulcer [38]. System analysis of metabolic networks that are a central paradigm in biology will help us in identifying new drug targets which in turn will generate more in-depth understanding of the gastric ulcer mechanism and thus provide better guidance for drug discovery.

## Conclusion

The potential application of systems biology in medicine is infinite and will have a significant impact on TCM, clinical research and drug development. Metabolomics represents an emerging and powerful discipline that provides an accurate and dynamic picture of the phenotype of biosystems through the study of potential biomarkers of gastric ulcer that could be used for therapeutic targets and discovery of new drugs. In this study, for the first time, we report a comprehensive analysis of metabolic patterns of the treatment of acid-induced gastric ulcer with CA. The action mechanism of CA was analyzed by an effective approach of metabolite profiling, and we have identified 10 differential metabolites associated with gastric ulcer. More importantly, according to the 10 differential metabolites, 7 related pathways were discovered. Particularly, fatty acid metabolism and sphingolipid metabolism were found as the most altered functional pathways associated with gastric ulcer according to related gene epression analysis. Compared with the alterations of gastric ulcer related metabolites, most of them were reset to a healthier level after CA administration. Our findings also show that CA exhibited preventive efficacy against gastric ulcer by adjusting these multiple metabolic pathways to their normal state and may be mediated through protein, gene, enzyme, and bioprocess. Based on our findings, this makes these pathways possible therapeutic targets for advanced gastric ulcer. In conclusion, the results contribute to a further understanding of gastric ulcer mechanisms. In addition, this study of potential metabolites could be used to achieve multiple targets for treatment of gastric ulcer, that lay foundation for finding therapeutic targets and discovering new multi-target drugs.
